# Find Joy in Sorrows: The Role of Hope in Buffering the Psychological Impact of COVID-19 on Chinese University Students in Hong Kong

**DOI:** 10.3390/bs13100821

**Published:** 2023-10-05

**Authors:** Stephen Cheong Yu Chan, Qi Lu Huang, Andrew Yiu Tsang Low

**Affiliations:** 1 Felizberta Lo Padilla Tong School of Social Sciences, Caritas Institute of Higher Education, Hong Kong; alow@cihe.edu.hk; 2 Department of Social and Behavioural Sciences, City University of Hong Kong, Tat Chee Avenue, Kowloon, Hong Kong; qlhuang5-c@my.cityu.edu.hk

**Keywords:** Chinese young adults, hope, agency thinking, positive emotions, subjective wellbeing, COVID-19

## Abstract

The global coronavirus disease 2019 (COVID-19) crisis has exerted significant psychological impacts on university students who have faced drastic changes in the learning mode and suspension of classes. Despite these challenges, many students maintained subjective well-being. In this study, we examined the role of “hope” as a potential protector to maintain their subjective well-being when facing adversity during this global crisis. Specifically, we explored the mediating role of two hope components (agency thinking and pathways thinking) on the association of positive emotions and life satisfaction among Chinese university students. We conducted an online survey at a local university and recruited a total of 315 undergraduates from the humanities, creative arts, and social sciences programs through convenience sampling. Participants confirmed their informed consent and completed a set of self-administered questionnaires measuring positive emotions, hope, life satisfaction, and demographic variables. The results of mediation testing indicated that, during a global crisis such as COVID-19, positive emotions indirectly influenced life satisfaction through agency thinking rather than pathways thinking. The findings highlight the importance of agency thinking among Chinese university students in adverse situations and provide valuable insights for psychological interventions during a crisis. The article concludes by discussing possible explanations and implications of the findings in a post-pandemic world.

## 1. Introduction

The introduction of quarantine and stay-at-home orders during the COVID-19 pandemic increased university students’ anxiety, stress, and frustration. They were less satisfied with their lives than before the pandemic (Ammar et al., 2020; Soest et al., 2020) [1,2]. The daily routine of most university students was disrupted by the closure of schools and the replacement of face-to-face with online teaching. These changes introduced various stressors into students’ daily lives and studies. Students experienced much uncertainty when they needed to accommodate new learning and assessment methods (Jung et al., 2021) [3]. In addition, the closure of physical venues restricted their social interactions and social activities with peers and classmates, an essential developmental task for young adult development. Students’ restricted social lives during the pandemic generated loneliness, worsened mental health and well-being, and an increase in depression and anxiety (Odriozola-González et al., 2020; Padron et al., 2021) [4,5]. Students were concerned about academic delays due to COVID-19 and worried about social support, factors related to increased levels of anxiety (Dhar et al., 2020) [6]. However, there has been little research into Chinese Hong Kong university students’ satisfaction with life during this period, and the variables mediating their life satisfaction have not been explored. An international study indicated that university students’ capacity to increase their psychological well-being and satisfaction with life while also adopting adaptive coping strategies could alleviate these negative impacts (Lopes and Nihei, 2021) [7]. Given the research gaps, this study investigated the relationship between life satisfaction and COVID-19 among university students in Hong Kong, with a particular focus on the mediating role of “hope”.

### 1.1. Mental Health among University Students in Hong Kong

University students face the transition from adolescence to adulthood and from high school to university. They are subject to considerable demands to meet these new roles and adjust to their new stage of life. There are new academic demands, uncertainty about their future careers and professional development, and new interpersonal relationships, along with stress, uncertainty, and anxiety, all of which challenge students. International studies indicated that university students, e.g., medical students, were at an increased risk of depression during COVID-19 compared with non-medical university students, especially women [8,9]. Another study recently in Italy indicated that the dysfunctional cognitive thinking style “All or nothing” was a significant predictor of traumatic psychological distress. In this study, the optimistic thinking style of a quarter of the examined students was negatively correlated with psychological distress [10]. A study in Hong Kong indicated that more than 30% of university students suffered from depression, over 40% from anxiety, and around 20% from distress (Li et al., 2021) [11]. A 2018 study indicated that over 50% of university students in Hong Kong had symptoms of depression and anxiety (Lun et al., 2018) [12]. Of these, 9% reported severe depressive symptoms, and 5.8% had symptoms of severe anxiety. Symptoms of depression and anxiety among university students have been correlated with suicidal ideation, reduced academic ability, and adverse effects on their family and social relationships. Various studies indicate that life satisfaction is related to successful adaptation to life and is negatively associated with depression and anxiety (Yang et al., 2018) [13]. Life satisfaction is positively associated with self-efficacy, social support, and gratitude. Therefore, life satisfaction is crucial for university students facing developmental challenges and unusual periods such as COVID-19. However, the life satisfaction of university students in Hong Kong during COVID-19 has been rarely explored.

### 1.2. Hope as a Reliable Source of Resilience

Hope is defined as the psychological state of a person who has clear and conscious goals towards which they are striving (Eliott, 2005) [14]. To be hopeful, a person develops a trait belief in which they have a determination (agency thinking) and a plan to meet the goal successfully (pathways thinking). Specifically, agency thinking pertains to an individual’s perceived ability to start and maintain progress along certain routes to achieve their objectives, while pathways thinking is the individual’s ability to generate several potential paths toward goal attainment. Hope has been positively associated with various domains of life, such as academic success (Marques et al., 2017) [15], physical health (Schiavon et al., 2017) [16], psychological adjustment (Yeung et al., 2015) [17], and subjective well-being (Pleeging et al., 2021) [18]. It is negatively associated with psychological distress, including depression and anxiety (Arnau et al., 2007; Rustøen et al., 2010) [19,20]. In school settings, Snyder and colleagues (1996) revealed that hope was positively correlated with positive thoughts and negatively correlated with negative thoughts among college students [21]. As Snyder et al. (2018) suggest [22], it is crucial to consider both components of hopeful thinking, in which one’s hope level is positively related to both agency and pathways thinking. In other words, hopeful individuals, even under challenging life conditions, believe they can find alternative solutions and are motivated to apply them.

Furthermore, hope is regarded as a reliable source of resilience with various benefits (e.g., better adjustment and quality of life) (Long et al., 2020) [23]. Studies have revealed hope as a mediator between maladaptive variables (e.g., psychological vulnerability) and adaptive variables (e.g., life satisfaction: Rustøen et al., 2010; Satici, 2016) [20,24]. In an integrative review of university students’ mental health (Griggs, 2017) [25], hope was related to improved coping with life challenges and better well-being. It can also protect students from suicide and self-deprecatory thinking. This suggests that high-hope individuals may regain their motivation faster, create more alternative routes to their desired goals than low-hope individuals (Long and Gallagher, 2018) [26] and use more effective coping strategies to tackle uncontrollable circumstances (Lee and Gallagher, 2018) [27] when facing stressors or obstacles. This leads to better psychological adjustment and well-being (Snyder et al., 2018) [22]. Therefore, these findings support the notion that hope can play a significant role in dealing with various life challenges and enhancing one’s subjective well-being, and it is crucial to understand the mechanism of its effect.

### 1.3. Exploring the Dimensions of Hope: Differential Effects of Agency and Pathways Thinking

There are several components of “hope”. According to Snyder’s hope theory (1995; 2002) [28,29], the key components of hope are a person’s ability and flexibility to develop different pathways to achieve their goals and their ability to overcome difficulties in achieving those goals. Thus, agency thinking and pathways thinking share the contribution of hope (Snyder, 2002) [29]. To view hope this way is unidimensional. Recent studies indicate some scholars’ contribution to considering hope as a multidimensional construct, and a two-factor model of hope was identified among college students (Babyak et al., 1993) [30]. Recent studies (Chan and Huang, 2022; Tong et al., 2010) [31,32] have observed that agency thinking and pathways thinking have varying effects on different outcomes. For example, a dominant role for agency thinking in goal pursuit and actual performance was discernible among university students, while in the same sample, pathways thinking was not relevant to goal pursuit (Crane, 2014) [33].

### 1.4. Positive Emotions, Hope and Life Satisfaction: Application of the Broaden-and-Build Model

According to the broaden-and-build model, positive emotions (e.g., joy and contentment) can broaden thought–action repertoires (e.g., cognition resources) and build psychological resilience to help promote positive subjective well-being (Li et al., 2022) [34]. It is suggested that when individuals focus on achieving goals, experiencing positive emotions could boost their anticipation regarding their ability to achieve goals successfully (agency thinking) and foster their capability to initiate various ways of pursuing goals (pathways thinking). To understand the role of hope in promoting resilience and subjective well-being, Chang, Chang, and Kamble (2019) [35] applied Fredrickson’s (1998, 2001) [36,37] broaden-and-build model to examine the potential mediating role of hope between positive affectivity and life satisfaction. Studies have revealed that high-hope individuals possess several attributes that make them more satisfied. These include more creativity (Namono et al., 2021) [38], more persistence in pursuing their goals (Gallagher et al., 2017) [39], more ability to see opportunities and ways to achieve their desired goals (Pleeging et al., 2021) [18], and more resilience when facing negative situations (Arampatzi et al., 2020) [40]. Studies have shown that positive emotions can lead to both hope cognitions (agency and pathways thinking) (Ciarrochi et al., 2015 [41]) that might improve psychological outcomes (e.g., life satisfaction) by building hope agency and/or expanding hope pathways through broadening an individual’s thought–action repertoire.

Previous studies (e.g., Chang, Chang, Li, et al., 2019) proposed that the two components of hope might have different impacts on psychological outcomes [42]. For example, research has demonstrated that agency thinking and pathways thinking exhibit a positive correlation with life satisfaction across cultures, including Western (e.g., European Americans) and Eastern groups (e.g., Asian Indians and Chinese). However, the association between positive emotions and life satisfaction was mediated only by agency thinking but not pathways thinking, and the full mediation was found among Eastern groups but not in Western samples. This might underscore the relative importance of building agency or pathways thinking in enhancing the effect of positive emotions on life satisfaction under different circumstances.

### 1.5. Positive Emotions and Life Satisfaction

Positive emotions are consistently correlated with higher life satisfaction (Cohn et al., 2009) [43]. In a meta-analysis of experiments assessing the causal relationship between positive emotion and adaptive behavior in adults, Lyubomirsky et al. (2005) found that eliciting positive emotions might lead to increased creativity and flexible thinking [44]. Consistent with the notion of broaden-and-build theory (Fredrickson, 2001) [37], positive emotions help people to think of more ways to cope with problems (Fredrickson and Joiner, 2002) [45], build lasting resources, and buffer the effects of crises (Fredrickson and Cohn, 2008) [46], helping people to feel happier and more satisfied with life.

### 1.6. Hope and Life Satisfaction

The positive relationship between hope and life satisfaction has been found among various age groups (Bronk et al., 2009; Raats et al., 2019) and cultural groups (Chang, Chang, and Kamble, 2019) [35,47,48]. Research findings indicate that hopeful people may have positive expectations for the future, their lives are more satisfying, and they are more content (Hassan et al., 2018) [49]. Specifically, compared to pathways thinking, agency thinking predicts life satisfaction better (Bailey et al., 2007) [50]. Bailey et al. argued that people’s subjective belief that they can achieve their desired goals might lead to better subjective well-being, rather than a belief that they can generate effective means to overcome crises and obstacles [50].

### 1.7. Hope as a Protective Factor in a Global Health Crisis

Since the World Health Organization (WHO) declared the novel coronavirus (COVID-19) outbreak a pandemic, numerous studies have investigated its impact on physical and subjective well-being (Cao et al., 2020; Choi et al., 2020) [51,52]. Some scholars have also identified potential resiliency factors during stressful events such as the COVID-19 pandemic, reporting hope’s association with higher resilience to stressors, lower psychological distress, and better subjective well-being (Gallagher et al., 2021; Genç and Arslan, 2021) [53,54]. In this respect, hope could be a source of resiliency that promotes life satisfaction, particularly during times of stress and hardship (e.g., a global health crisis; Gallagher and Lopez, 2009 [55]).

In recent years, scholars have suggested that hope is a flexible construct that can be cultivated deliberately (Cheavens et al., 2006; Feldman and Dreher, 2012) [56,57]. This concept has led to the implementation of hope-enhancement tactics in psychotherapy and interventions, such as hope therapy (Lopez et al., 2018) and the Brief Hope Intervention (Chan et al., 2019) [58,59], aiming to boost individuals’ hopefulness and well-being. In this study, we were also keen to explore the influence of hope in the context of the pandemic. If our findings establish a positive correlation between hope and improved subjective well-being during this crisis, it would underscore the value of incorporating more hope-based programs or interventions. Such initiatives would provide individuals with essential tools to manage better and overcome challenges and adversity.

### 1.8. Purpose of the Present Study

The current study addresses the research gap regarding life satisfaction levels among Chinese university students in Hong Kong during the COVID-19 pandemic. It further enriches the global body of literature on the interplay between positive emotions and hope, particularly regarding agency thinking and pathways thinking and their mediating role in life satisfaction among university students in Hong Kong. We investigated the potential mediating roles of both agency and pathways thinking in elucidating the relationship between positive emotions and life satisfaction. As suggested in our literature review, we hypothesized that agency thinking would assume a more substantial role than pathways thinking in mediating the connection between positive emotions and life satisfaction.

## 2. Materials and Methods

### 2.1. Participants and Procedures

University students studying in Hong Kong were invited to participate in an online survey. Ethical approval was obtained from the university’s research ethics committee in accordance with the guidelines and procedures for ethical review regarding human research. After gaining permission from the participating university’s School of Arts and Social Sciences, the invitation was sent to students via their institution’s e-mail with an online survey link. Data were collected through a convenience sampling approach between February and July 2021. With the help of the teaching staff, over 500 e-mails were sent to the students enrolled in humanities, creative arts, and social sciences programs, and 315 completed questionnaires were returned. Before accessing the online survey on the platform Qualtrics, respondents were asked to read the purpose and procedures of the study in the consent form. Respondents were reassured that their participation was voluntary, and informed consent was obtained before initiating the questionnaire. They were also made aware of their right to withdraw from the study at any time, with the assurance that their information would be handled with the utmost confidentiality and their identities would remain anonymous.

### 2.2. Measures

#### 2.2.1. Positive Affect

The Positive and Negative Affect Schedule (PANAS; Thompson, 2007) was administered to measure an individual’s affect [60]. The scale comprises 20 items: ten items quantifying positive affect, demonstrated through feelings, such as “interest” and “excitement”, and ten items assessing negative affect, demonstrated through emotions such as “distress” and “guilt”. Using a scale from 1 = “very slightly” to 5 = “extremely”, respondents rated each item by considering how they experienced a particular mood at a specific time. The total score of each affect was added and averaged, with higher scores representing higher levels of positive or negative affect. The Chinese version of PANAS has been validated, showing satisfactory reliability, and has been used in the Chinese college population (Li et al., 2020) [61]. In this study, only the PANAS-Positive (PANAS-P) items were used for data analysis, and Cronbach’s alpha was 0.88.

#### 2.2.2. Hope

The 12-item Dispositional Hope Scale (DHS) was selected to assess hope (Snyder et al., 1991) [62], with eight items measuring hope and four filter items. DHS measures two components of hope: agency thinking (AT) e.g., “I energetically pursue my goals”) and pathways thinking (PT), e.g., “There are lots of ways around any problem”). The filter items were neglected. Respondents were asked to rate each statement on an 8-point Likert-type scale, ranging from 1 = “definitely false” to 8 = “definitely true”. The Chinese version of DHS has been validated and used for Chinese college students (Chang, Chang, Li, et al., 2019) [42]. In this sample, Cronbach’s alphas for agency thinking and pathways thinking were 0.81 and 0.82, respectively. Responses for each component were averaged as a mean score, with higher scores on the DHS indicating a greater sense of hope.

#### 2.2.3. Life Satisfaction

The Satisfaction with Life Scale (SWLS) was used to measure the cognitive component of subjective well-being (Diener et al., 1985) [63]. The SWLS comprises five statements, and respondents are asked to rate each item on a 7-point Likert-type scale, ranging from 1 = “strongly disagree” to 7 = “strongly agree”. Sample SWLS items include ‘I am satisfied with my life’. A higher score on the SWLS represents a greater level of satisfaction with life. Cronbach’s alpha for SWLS was 0.86.

#### 2.2.4. Sociodemographic Variables

Sociodemographic variables, including age, sex, monthly income level, and financial satisfaction, were collected. Monthly income was categorized into two options: 0 = “HKD 0 to HKD 5999” and 1 = “above HKD 6000”, and respondents’ perceived adequacy of their personal finances (financial satisfaction) was measured using a 5-point Likert scale. This scale ranges from “1”, signifying “extremely inadequate”, to “5”, indicating “highly adequate”. The objective of this measurement is to gauge respondents’ personal evaluation of their financial sufficiency compared to their spending habits (Garrett and James III, 2007) [64].

### 2.3. Analytic Procedures

Descriptive statistics were processed for all sociodemographic variables and target variables. Correlation analyses were undertaken to explore the relationships between sociodemographic variables (PANAS-P, AT, PT, and SWLS). The variation inflation factor (VIF) was used to examine any collinearity between variables in the regression model. All VIF values were lower than five, suggesting no multicollinearity among variables in this study. The parallel mediation model was assessed by choosing PANAS-P as the predictor variable, AT and PT as mediating variables, and SWLS as the dependent variable. The parallel mediation analyses were performed using the SPSS macro-PROCESS (model 4; Hayes, 2013) [65]. The bias-corrected bootstrapping method was conducted to examine the indirect effects based on 10,000 bootstrap samples at a 95% confidence interval. If the mediating variables with the bias-corrected confidence interval did not contain a zero, the indirect effects were taken as statistically significant, and mediation was established. Total effects and direct effects were also calculated.

## 3. Results

### 3.1. Descriptive Statistics

Table 1 presents the demographic information of all respondents. The sample had a mean age of 22.08 (SD = 2.74) and comprised 211 female participants (67.00%) and 104 male participants (33.00%). Approximately 77% earned less than HKD 6000 per month. The average financial satisfaction score was 3.16 (SD = 0.99), indicating that respondents felt they had adequate financial resources.

Table 2 shows the correlations between all measures and SWLS. Specifically, financial satisfaction was significantly positively correlated with PANAS-P (r = 0.13, *p* = 0.019), AT (r = 0.16, *p* = 0.004), PT (r = 0.15, *p* = 0.007), and SWLS (r = 0.31, *p* < 0.001). In general, PANAS-P, AT, PT, and SWLS were moderately or strongly correlated (ps < 0.001).

### 3.2. Parallel Mediation Statistics

With reference to the results of the correlation analysis, only financial satisfaction was associated with SWLS and was treated as the covariate in the mediation analyses. We ran a parallel mediation model to examine the relationships between positive emotions and life satisfaction as mediated by agency and pathways thinking among respondents. 

**Figure 1:**
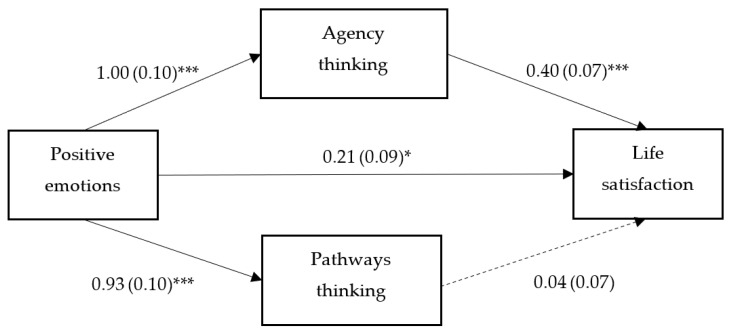
Outcomes from the analysis assessing the mediating roles of agency and pathways thinking in the relationship between positive emotions and life satisfaction among Chinese university students. All figures depicted are non-standardized regression coefficients along with their respective standard errors. *N* = 315. * *p* < 0.05, *** *p* < 0.001.

PANAS-P was significantly associated with AT (B = 1.00, *p* < 0.001, 95% CI = 0.81, 1.20) and PT (B = 0.93, *p* < 0.001, 95% CI = 0.73, 1.12). It was also associated with SWLS (B = 0.21, *p* = 0.021, 95% CI = 0.33, 0.39). Only AT was further positively associated with SWLS (B = 0.40, *p* < 0.001, 95% CI = 0.28, 0.53). As a significant direct effect was found (shown in Figure 1), AT partially mediated the relationship between PANAS-P and SWLS (ab = 0.41, *p* < 0.001, 95% CI = 0.26, 0.56).

## 4. Discussion

### 4.1. Investigating the Associations: Positive Emotions, Hope, and Life Satisfaction 

This study investigated whether positive emotions had any association with Chinese university students’ agency thinking and pathways thinking and whether this association was related to their life satisfaction during the global COVID-19 crisis. Echoing the findings of previous studies, significant positive associations were found between positive emotions, hope components, and life satisfaction.

In particular, in line with the broaden-and-build model (Fredrickson and Branigan, 2005) [66], our findings supported the notion that positive emotions could both widen the array of positive thoughts and build thought–action repertoires. That is, positive emotions were positively associated with both agency and pathways thinking. Experiencing more positive emotions might help university students sustain positive thoughts, initiate actions toward goals, and generate possibilities for achieving goals.

Interestingly, although all the variables were positively associated, positive emotions were generally moderately correlated with agency and pathways thinking (rs~0.50), and the magnitude of the associations between positive emotions and life satisfaction was relatively weaker (rs~0.40). This might suggest that hope could be a potential mediator between the above relationships.

### 4.2. Distinct Mediating Role of Hope between Positive Emotions and Subjective Well-Being

This study also investigated the role of hope as a mediator of the associations between positive emotions and life satisfaction after controlling for the covariate. Our mediation analyses indicated a distinct difference in the mediating role of hope components. Specifically, agency thinking, but not pathways thinking, fully mediated the relationship between positive emotions and life satisfaction. Based on the differences drawn by the mediation models, we may argue that although agency thinking and pathways thinking are two reciprocal components of the hope construct, they may each play a specific role in contributing to different outcomes of subjective well-being. 

In our study, students who experienced frequent positive emotions might not necessarily feel satisfied with life until they could build a variety of personal resources (Fredrickson, 1998) [36]. With more personal sources initiated by positive emotions, individuals could establish their will and understand ways to reach their goals, which in turn enhanced their well-being (Cohn et al., 2009) [43]. Our results are consistent with previous studies; the partial mediation model consolidated the robust role of agency thinking relative to pathways thinking between positive emotions and life satisfaction (Chang, Chang, and Kamble, 2019; Chang, Chang, Li, et al., 2019) [35,42].

### 4.3. Implications for Psychotherapy and Intervention in the Post-Pandemic Era

Hope has been a cornerstone in positive psychology for a long time, recognized as a protective element that can reduce vulnerability and foster resilience in the face of life’s crises (Snyder, 2002) [67]. The adaptability of hope, as a trait, has been integrated into psychotherapy and intervention strategies aimed at promoting additional hopeful thinking, adaptability when dealing with challenges, and proactive problem-solving.

Several intervention initiatives and procedures, such as community-based prevention programs for suicide and brief interventions for cancer patients, have been designed to boost agency and pathways thinking during crises and adversity (Chan et al., 2019; Huen et al., 2015) [59,68]. Cognitive-behavioral therapy, in particular, is a structured, time-bound, problem-centered, and goal-oriented form of psychotherapy. This therapeutic approach focuses on augmenting clients’ awareness of their thoughts, feelings, and behaviors. By nurturing clients’ self-confidence and their ability to attain personal goals, cognitive-behavioral therapy strengthens agency thinking—a key component of hope. This form of therapy can benefit clients grappling with suicidal thoughts or depression symptoms by enhancing their overall level of hope.

Regarding the pandemic’s negative impact, about a quarter of respondents reported worsening mental health during the COVID-19 pandemic (Choi et al., 2020) [52]. Therefore, supporting young people with practical strategies to promote active coping is important. The study’s findings primarily point to hope’s role in protecting life satisfaction from the adverse impact of COVID-19. It reflects a potential benefit to mental health (e.g., life satisfaction) through instilling and strengthening hope, as suggested by Snyder (1995) [28].

While the impact of the COVID-19 pandemic appears to be slowly easing globally, it is undeniable that the crisis may have long-lasting effects. University students, in particular, bear significant psychological and social burdens, as they are often expected to abruptly transition between online and in-person learning, leading to heightened stress levels (Wang et al., 2021; Waters et al., 2021) [69,70]. 

In the face of such challenges, developing a hopeful mindset can serve as a powerful coping tool, facilitating better management of these changes. This mindset not only promotes better mental health but also enhances academic performance (Long and Gallagher, 2018) [26]. This study underscores the significance of focusing on Student Counseling Services as a valuable strategy to assist university students in coping with emotional distress and pursuing goals with higher confidence and more ways to tackle obstacles (Pedrotti et al., 2008) [71]. Ultimately, this may reduce the potential for psychopathological profiling over time. Besides individual counselling, many effective Western-developed programs tried to integrate hope concepts into the intervention, such as Lopez’s “Making Hope Happen” (2013) [72]. It is imperative to further investigate the effectiveness of these university services in promoting students’ mental health, especially in the post-pandemic era, which may contribute to Goal 3: Good health and well-being, one of the Global Goals of the 2030 Agenda for Sustainable Development. Such services should aim to harness the power of hope, especially pathways thinking, thus bolstering university students’ emotional well-being. 

### 4.4. Limitations and Future Studies

This study suggests distinct pathways between positive emotions, hope, and life satisfaction among Chinese university students during COVID-19. However, several limitations need to be considered. First, this study adopted a cross-sectional design; thus, causality could not be confirmed. 

Second, since our study was conducted during the COVID-19 period to capture the situation of respondents during a global crisis, a very large sample size cannot be guaranteed. Comparisons among different populations may give a better picture of any differences between university students and other young adults who were not studying at university. In addition, the study could not consider the developmental processes that young adults may encounter in their lives. For example, a six-year longitudinal study found a declining trend in life satisfaction and a gradually increasing trend in hopelessness among Chinese adolescents in Hong Kong (Shek and Liang, 2018) [73]. This may be because adolescents face more challenges over time, making them more stressed. Therefore, a longitudinal design is recommended to consolidate the effects and changes of the potential mediating role of hope components in accounting for the relationship between positive emotions and life satisfaction.

Third, our participants were solely recruited from a university in Hong Kong through convenience sampling, and all of them are Chinese undergraduates. Due to the sampling method and the nature of the sample, we should be cautious when generalizing the study’s results to the experiences of other ethnic groups. Previous findings have shown a possibility of greater involvement of hope agency over hope pathways in other racial and cultural groups (Chang, Chang, Li, et al., 2019) [42]. Future studies should include diversity and explore mechanisms across ethnic groups to provide appropriate interventions for specific populations.

## 5. Conclusions

This study investigated the potential mediating influence of “hope”—specifically focusing on the component of agency thinking—in the relationship between positive emotions and life satisfaction among Chinese university students during the COVID-19 pandemic. Our findings demonstrate that, despite facing drastic changes in learning modes and class suspensions, agency thinking played a significant role in amplifying the influence of positive emotions on subjective well-being. This underscores the importance of agency thinking in fostering resilience among students in the face of adversity. These insights are invaluable for developing psychological interventions to enhance mental health during a major crisis. For future research, we suggest a longitudinal study design to further validate and expand these findings, emphasizing the potential role of hope in mitigating the psychological impacts of a crisis such as the COVID-19 pandemic.

## Figures and Tables

**Table 1:** Descriptive statistics.

	Overall Sample (*N* = 315)
	Frequency (%)/Mean (*SD*)
Variables	
Age (years)	22.08 (2.74)
Sex	
Male	104 (33%)
Female	211 (67%)
Major Disciplines ^#^	
Creative Arts	69 (25%)
Humanities and Language	71 (26%)
Social Sciences	138 (49%)
Year Standing ^#^	
Year 1	45 (16%)
Year 2	76 (27%)
Year 3	69 (25%)
Year 4	88 (32%)
Monthly Income (HKD)	
<HKD 6000	243
HKD 6000 or above	72
Financial Satisfaction	3.16 (0.99)
Hope—Agency Thinking	4.82 (1.37)
Hope—Pathways Thinking	5.20 (1.33)
PANAS-Positive	2.74 (0.67)
Life Satisfaction	3.87 (1.20)

*Note*. ^#^ Number of valid responses was 278.

**Table 2:** Correction table among all measures with SWLS.

	Sex	Income	Financial Satisfaction	PANAS-P	AT	PT	SWLS
Age	−0.08	0.40 **	−0.15 *	0.03	0.08	0.10	0.02
Sex		−0.18 **	0.15 **	−0.13 *	−0.06	−0.04	0.03
Income			0.02	0.06	0.13 *	0.10	0.00
Financial Satisfaction				0.13 *	0.16 **	0.15 **	0.31 ***
PANAS-P					0.51 ***	0.48 ***	0.40 ***
AT						0.80 ***	0.59 ***
PT							0.50 ***

* *p* < 0.05, ** *p* < 0.01, *** *p* < 0.001.

## Data Availability

The data presented in this study are available on request from the corresponding author.
